# CNN-based flow field prediction for bus aerodynamics analysis

**DOI:** 10.1038/s41598-023-48419-4

**Published:** 2023-12-01

**Authors:** Roberto Garcia-Fernandez, Koldo Portal-Porras, Oscar Irigaray, Zugatz Ansa, Unai Fernandez-Gamiz

**Affiliations:** 1 Nuclear Engineering and Fluid Mechanics Department, University of the Basque Country, UPV/EHU, Nieves Cano 12, Vitoria-Gasteiz, 01006 Araba, Spain; 2 Sunsundegui S.A., Polígono Ibarrea, s/n, 31800 Altsasu, Navarra Spain

**Keywords:** Mechanical engineering, Fluid dynamics

## Abstract

The aim of this article is to evaluate the ability of a convolutional neural network (CNN) to predict velocity and pressure aerodynamic fields in heavy vehicles. For training and testing the developed CNN, various CFD simulations of three different vehicle geometries have been conducted, considering the RANS-based k-ω SST turbulent model. Two geometries correspond to the SC7 and SC5 coach models of the bus manufacturer SUNSUNDEGUI and the third one corresponds to Ahmed body. By generating different variants of these three geometries, a large number of representations of the velocity and pressure fields are obtained that will be used to train, verify, and evaluate the convolutional neural network. To improve the accuracy of the CNN, the field representations obtained are discretized as a function of the expected velocity gradient, so that in the areas where there is a greater variation in velocity, the corresponding neuron is smaller. The results show good agreement between numerical results and CNN predictions, being the CNN able to accurately represent the velocity and pressure fields with very low errors. Additionally, a substantial improvement in the computational time needed for each simulation is appreciated, reducing it by four orders of magnitude.

## Introduction

The consideration of aerodynamic effects in the design of heavy vehicles has been a field of research and technical development in recent years, due to the importance of fuel price in operating costs, the requirements imposed by the European decarbonization policies and the introduction of the electrification of engines. The influence of the aerodynamic drag coefficient on these three factors is evident, since as point out by Ansa et al.^1^, a lowered drag results in decreased consumption, reduced emissions of polluting gases and increased range of use of electric vehicles, reducing the carbon footprint throughout its useful life. These technical advances have been successfully applied in the transport of goods, since aerodynamic improvements are easy to transfer from one model to another, due to the uniformity and similarity between these vehicles. However, these aerodynamic improvements are not immediately transferable to other types of heavy vehicles such as coaches. Therefore, their own aerodynamic improvements must be developed.

As for computational development, in recent years, the evolution of calculation hardware, together with improvements in fluid dynamic analysis software have managed to considerably increase the precision of CFD results. However, access to these computational tools carries a high cost, firstly economic, access to compute clusters or GPU processors is very limited due to their high price, secondly, despite performance improvements and the power of the equipment, the time required to obtain a precise solution is still very high, and thirdly, the complexity of the physics and mathematics associated with a correct understanding of the problem. This high cost means that the use of fluid dynamic analysis tools is limited to large manufacturers. All these issues have led researchers to look into alternative flow-characteristic prediction methods. Many authors have applied Deep Learning as an addition to numerical fluid dynamics simulations as a way of optimizing results, by identifying potential mesh refinement areas or analyzing geometries. For example Ray and Hesthaven^2^ performed a study to identify cells where a result discontinuity was present, or Yan et al.^3^ who used machine learning techniques to optimize aerodynamic geometries.

One of the most recently improved methods is the use of accurately trained Convolutional Neural Networks (CNN) such as those developed by Portal-Porras et al.^4^ or Jacob et al.^5^. A data-driven surrogate model trained with high-accuracy CFD simulation-data can be used to develop aerodynamic improvements in an efficient manner. The development of a high-fidelity CNN serves for faster analysis of higher volumes of geometries over a broader area of design. This model allows designers to test and evaluate a higher number of variations over a shorter period of time than by using CFD solutions whilst maintaining a sufficient level of accuracy, providing a useful and complementary tool to CFD and Wind Tunnel testing.

This work builds upon Portal-Porras’s^4^ work on CNN implementation for aerodynamic device analysis purposes by adding deep learning (DL) solutions to large-vehicle design and optimization. Previous studies that implement similar solutions such as those conducted by Ye et al.^6^ (predicting pressure distributions around a cylinder based on the velocity-field and wake) or Ribeiro et al.^7^ (flow characteristic prediction for unsteady turbulent flows) show good agreement between the obtained results, CFD results and experimental data.

As for the prediction of drag coefficients using DL techniques, there are several different ways to go about it, as it is a more flexible estimation than that of a pressure or velocity field. Nevertheless, the use of CNNs for force coefficient prediction offers greater adaptability than other architectures, giving the user the ability to predict the drag coefficient of different geometries without a need of a greater alteration of the CNN as Zhang et al.^8^ points out.

On the field of data-driven methods, there are some approaches for vehicle aerodynamics, but it is still an unexplored field. For example, Misar et al.^9^ proposed a reduced order model (ROM) based on dynamic mode decomposition (DMD) to approach the flow around the Ahmed body and Mrosek et al.^10^ proposed another ROM based on proper orthogonal decomposition (POD) for vehicle aerodynamics, obtaining accurate results in both cases. Other authors use different deep learning methods. For example, Jaffar et al.^11^ used different machine learning techniques for drag force prediction of vehicles in platoon configuration, showing that artificial neural networks (ANNs) provide low-error predictions, and Mrosek et al.^12^ used a variational autoencoder (VAE) for field prediction around the DrivAer vehicle geometry, showing that it can outperform other ROMs.

Bayraktar et al.^13^ performed a study on the Ahmed body where an experimental test and a computational analysis were carried out and compared for a series of slant angles and their effect on lift and drag was studied along with the effect of the Reynolds number on both lift and drag forces. Additionally, Lienhart et al.^14^ created an accurate database under a defined set of conditions by using LDA (laser Doppler anemometry), HWA (hot-wire anemometry) and static pressure measurements on the flow field around and Ahmed body. The results obtained in these two studies are used for correlation of the CFD results used on this study as well as those used for the training of the CNN.

The main objective of this paper is the evaluation of the feasibility of predicting flow characteristics around heavy ground-vehicles by applying deep learning techniques and correlating results with CFD and experimental data, as well as the flow field prediction around the well-known Ahmed body geometry. On that note, velocity and pressure fields are predicted around different bus geometries and Ahmed body by means of a CNN developed to analyze unparametrized but similar geometries. In order to evaluate the performance of the CNN when predicting flow characteristics and the pressure and velocity fields, the error between the CNN predictions and the CFD numerical calculations has been estimated for a set of Ahmed bodies and bus geometries. It should be noted that this study is focused on the design and development of the CNN for the prediction of flow characteristics around heavy vehicles. For that reason, the CFD-based numerical methods used to obtain the dataset are based on simple models, avoiding high computational costs.

## Methodology

### Geometries

To carry out both the CFD and CNN studies, three main two-dimensional geometries have been used. Two bus models from the Sunsundegui bus manufacturer company, the model SC5 and SC7, and a scaled Ahmed Body.

As for the flow-domain dimensions, the Langley Full-Scale Tunnel was modeled to mimic the conditions displayed in Bayraktar et al.^13^. However, it was later scaled up by a factor of 12, to ensure the correct functioning of the CNN and make it, again, as similar as possible to the dimensions of the geometries of the buses. The bus and Ahmed body geometries combined with the domain defined according to the specified wind-tunnel dimensions amount to a blockage-ratio of around $BR=0.09$. Furthermore, the effect of the BR for ground vehicle simulations in open jet wind tunnels has been studied by Fu et al.^15^, concluding that increasing the BR yields and overprediction in drag compared with the free air drag. The larger the BR the greater the interference effect is, and therefore drag predictions are less accurate. The effect of BR must be considered in the comparison of CFD and experimental results for validation of the computational simulations. Considering the similarity between the computational domain BR and the WT BR in the Langley Full-Scale Tunnel, the defined domain is considered acceptable for the analysis of the Ahmed body and the bus geometries.

#### Ahmed body

**Figure 1:**
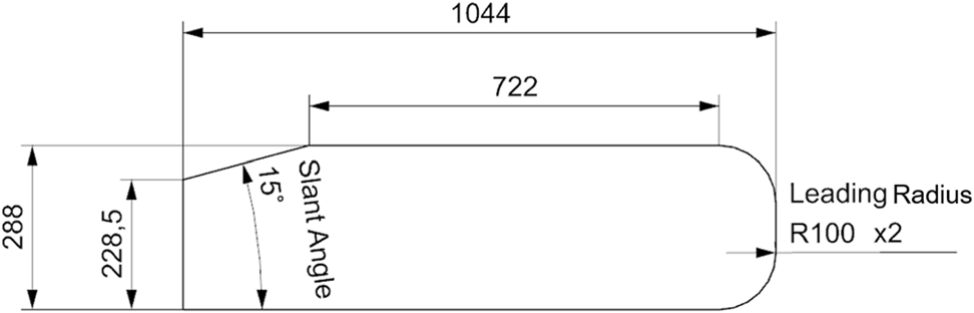
Ahmed body geometry.

The mentioned Ahmed body, shown in Fig. 1, is also known in literature as the Ahmed model, Ahmed et al.^16^. For this study the Ahmed body geometry has been scaled up, by a factor of 12, in order to make it as similar as possible to the bus in terms of dimensions, leading to an Ahmed body of 3,4 m of height and 12,5 m of length. This scale up is necessary in order to accurately train the convolutional neural network, since training geometries that differ too much in size will result in inaccuracies when predicting the velocity field around the test bodies.

To do so, 21 geometry variants have been drawn up to produce a wider range of training, validation and testing results for the CNN. The center-plane of these geometries were used. These alternatives consist on the variation of the original Ahmed body parameters. Specifically, the slant angle from 5° to 35°, with 5° increments, as well as the leading radii with values of 25 mm, 50 mm and 100 mm.

#### Sunsundegui SC5 and SC7

**Figure 2:**
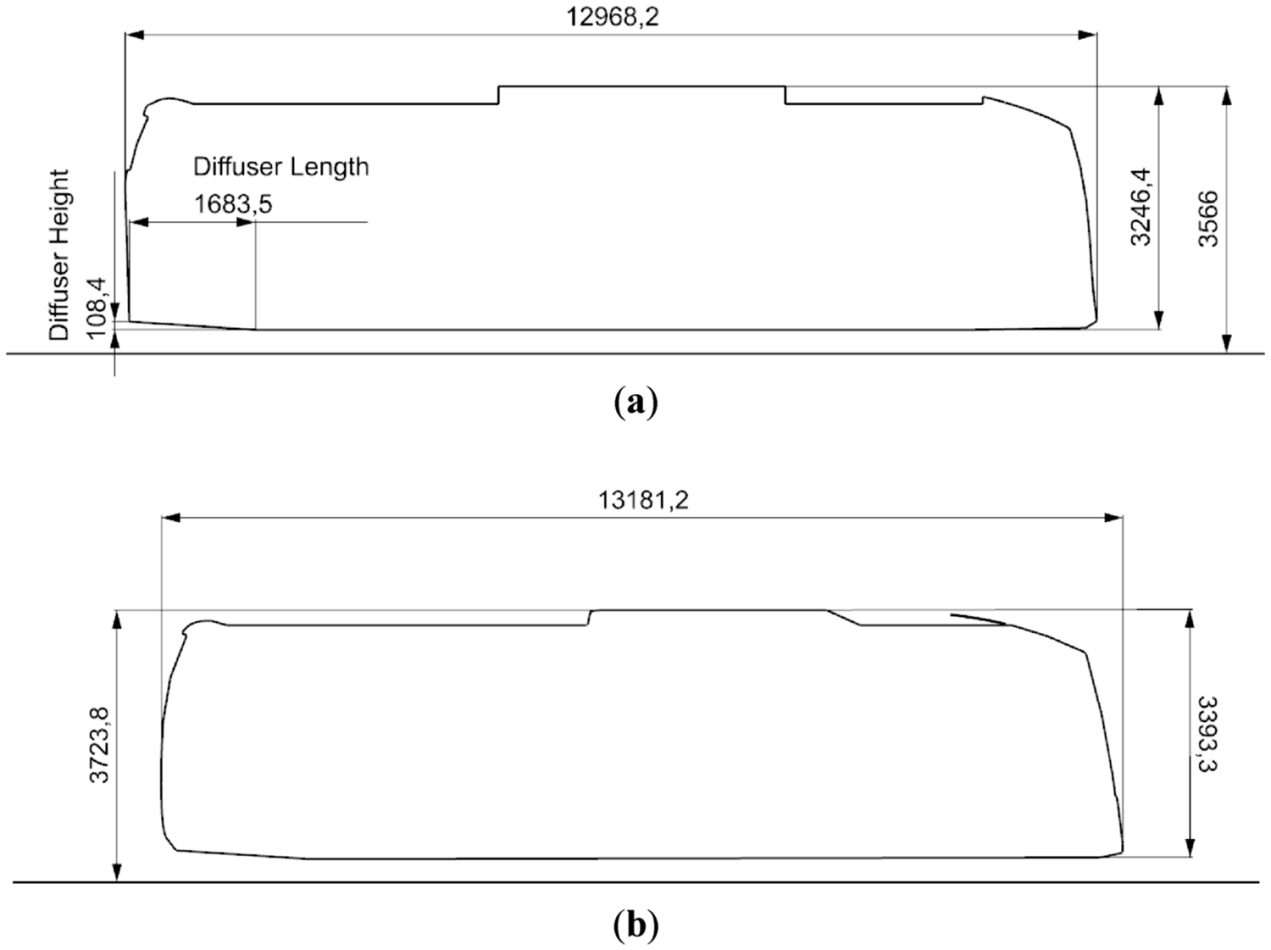
Bus geometry outlines (**a**) SC5, (**b**) SC7.

These two geometries (Sunsundegui SC5 and SC7) are cut longitudinally through the center plane of each vehicle to produce the two-dimensional bodies for the CNN. Figure 2a shows the geometry of the SC5 model and Fig. 2b the SC7 model. For the training, validation and testing of the CNN a total of 200 variations of the buses have been used. These geometry variations consist on the change of diffuser length and height. The different variants are shown in Table 1 and every possible combination of lengths and heights shown in this table is studied.

**Table 1:** Diffuser lengths and heights.

Diffuser lengths (mm)	Diffuser heights (mm)
1683 (original)	108.4 (original)
1200	30
1300	50
1400	70
1500	90
1600	110
1700	130
1800	150
1900	170
2000	190
2100	210

### Numerical set-up

In this section the set-up and conditions defined for the numerical simulations will be discussed and justified, so that the simulations fit the necessities for both the development of the CNN and the validation of the results in terms of aerodynamic drag and velocity and pressure fields.

The aforementioned 2D physical models were run under the same set of flow conditions using the commercial code Star-CCM+v2020.3.1^17^. These simulations were conducted for a constant-density, time-averaged steady state solution. As for the pressure–velocity coupling an upwind algorithm was used along with a linear upwind second order discretization scheme.

In terms of the boundary conditions set for the simulation (Table 2), the inlet velocity for all the simulations used for training, testing and validation of the CNN were set at 95 km/h for both the bus geometries and the Ahmed body cases. As for the result validation simulations a Reynolds number of around 4.3 M was defined to be able to compare the obtained values with those reported in the previously mentioned studies, ensuring as much independence as possible between the drag force and the Reynolds number. Additionally, a no-slip condition was defined for the ground but no moving ground was instated in the simulations with the goal of mimicking the conditions of the experiments conducted by Ahmed et al.^16^ rather than those seen on real life situations.

**Table 2:** CFD boundary conditions and configuration.

Air density	1.184 kg/m^3^
Dynamic viscosity	1.85508E−5 Pa s
Flow velocity	95 km/h
Turbulence model	k–ω SST
Convergence criteria	All residuals < 10^–3^ Standard deviation < 1 N for Drag over 100 iterations
Mesh type	2D quadrilateral Mesher
Prism layers	15 layers—thickness set for Y + < 1

#### Space discretization

For the discretization configuration a mesh dependency study was conducted by comparing the drag force coefficient obtained with 4 different meshes: coarse, medium, fine and finest (all of them refinements of the original coarse mesh) to ensure that the mesh has no significant effect on the results production.

The configuration of the initial mesh was done taking some basic considerations into account. Free stream flow cells were set at 2.5 m to reduce the computational power needed due to the low turbulence seen by these cells. Advanced cell-sizing controls are used to accurately represent the geometry as well as to capture the main flow structures around the body and wake of the vehicles. Additionally, in order to accurately resolve the boundary-layer and therefore the viscous and pressure forces around the body, a prism layer configuration has been used. 15 prism layers have been set at the body walls and the thickness has been defined to maintain a low wall y + of $Y+<1$ in order to ensure convergence.

**Figure 3:**
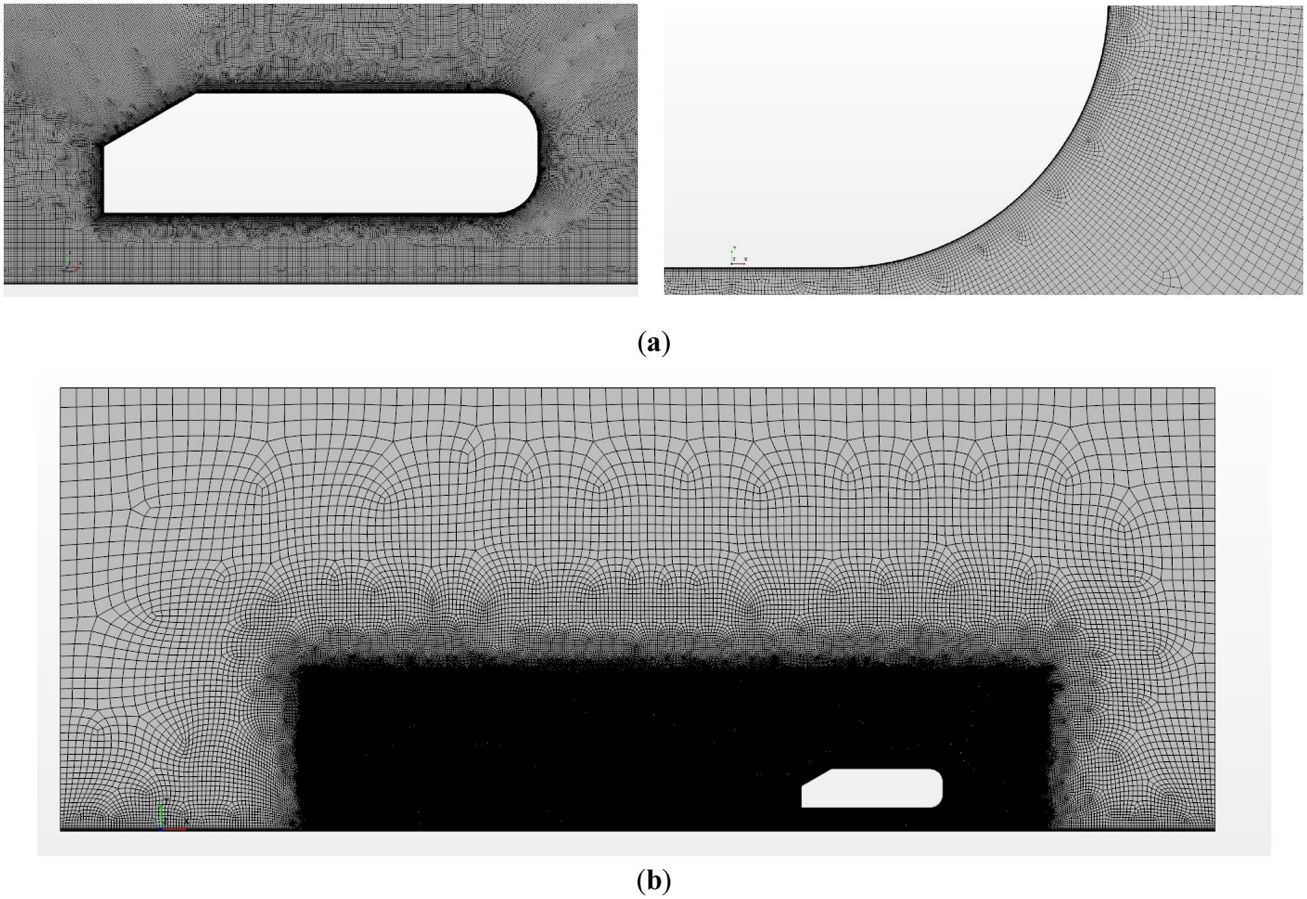
Fine mesh configuration (**a**) Ahmed body close up (**b**) whole domain.

The mesh sensitivity study results shown in Table 3 conclude that, since the main goal of this paper is the development of an accurate CNN able to replicate CFD results rather than experimental results, an error lower than 10% is considered as acceptable, and, in order to facilitate and speed up the generation of results for the training and testing of the CNN, a coarser mesh can be favorable. Therefore, the fine mesh configuration has been selected for the rest of the simulations. This Fine mesh can be seen in Fig. 3.

**Table 3:** Mesh independence study results.

	Number of cells	Cd	Error (%)
Coarse	57,000	0.415	9.78
Medium	107,000	0.411	8.73
Fine	455,000	0.397	5.02
Finest	1,463,000	0.395	4.49
Experimental	–	0.378	–

#### Turbulence model

Considering the Reynolds number and low Mach number at which ground vehicles, including buses, operate, the flow-field can be considered turbulent and incompressible. The boundary layer has been modelled as fully turbulent, with no laminar or transitional regions. In order to model the effects of turbulence on the mean flow, and on the flow gradients, the Reynolds averaged Navier–Stokes (RANS) equations require turbulence modelling.

The most widely used models, in the field of ground aerodynamics are the Eddy viscosity models (EVM) used in Reynolds averaged Navier–Stokes (RANS) approach which solve transport equations for certain turbulence controlling parameters with a lower computational cost compared to other physical and empirical methods such as the Large-Eddy simulation (LES) or direct numerical simulation (DNS) methods. Rodi^18^ proved RANS models to be more accurate than the direct numerical simulation (DNS) models for relatively higher Reynolds values (Re > 10^4^) on coarser grids. The RANS methods numerically solve the 3D time-averaged Navier–Stokes equations and time-averaged continuity equations and, approximate the unknown Reynolds stress tensor (RST) components.

These are divided into different turbulence solving methods/models. Depending on the application, several RANS models have been developed which represent different approaches on how to model the effects of the eddies in the BL also known as near wall behavior. Two-equation models such as, *k*–ε, add experimental based coefficients such as the “eddy-viscosity” coefficient (*C*μ). Eddy-viscosity models can be divided into two main groups: algebraic models, which are simple and cheap as they relate the eddy-viscosity to averaged-flow parameters for the outer region of the BL, and two-equations models, which solve the partial differential transport equations for two statistical-averaged scales (eddy length-scale/intensity-scale and velocity-scale generally). The two-equation models are far more exact and precise than the algebraic models but they require higher calculation costs as explained by Bradshaw^19^. Although, with the current computational developments solving this is achievable. The model used in this study is one of the most used two-equation models, due to its versatility and higher accuracy when resolving adverse pressure gradients: the k–ω SST (shear-stress transport) model developed by Menter^20^. This model derived from Wilcox’s^21^ original k–ω model. This model is able to blend, and eventually switch from the original k–ω model to Jones-Launder^22^ k–ϵ model using a wall-distance criterion based switching function, with the addition of accounting for the shear stress transport in adverse pressure gradients.

Menter’s^20^ k–ω SST model has been used for turbulence modelling and is defined with the following equations:

1 $$\frac{\partial \rho \kappa }{\partial t}+\frac{\partial {u}_{j}\kappa }{\partial {x}_{j}}={P}_{k}-{\beta }^{*}\rho \omega \kappa +\frac{\partial }{\partial {x}_{j}}\left[\left(\mu +{\sigma }_{\kappa }{\mu }_{t}\right)\frac{\partial \kappa }{\partial {x}_{j}}\right]$$

2 $$\frac{\partial \rho \omega }{\partial t}+\frac{\partial {u}_{j}\omega }{\partial {x}_{j}}={\gamma P}_{\omega }-{\beta }^{*}\rho {\omega }^{2}+2\left(1-{F}_{1}\right){\sigma }_{\omega 2}\frac{{u}_{t}}{\kappa }\frac{\partial \kappa }{\partial {x}_{j}}\frac{\partial \omega }{\partial {x}_{j}}+\frac{\partial }{\partial {x}_{j}}\left[\left(\mu +{\sigma }_{\omega }{\mu }_{t}\right)\frac{\partial \omega }{\partial {x}_{j}}\right]$$

where:

3 $${P}_{k}={u}_{t}\frac{\partial {u}_{i}}{\partial {x}_{j}}\left(\frac{\partial {u}_{i}}{\partial {x}_{j}}+\frac{\partial {u}_{j}}{\partial {x}_{i}}\right)-\frac{2}{3}\rho \kappa {\delta }_{ij}\frac{\partial {u}_{i}}{\partial {x}_{j}}$$

4 $${P}_{\omega }=\rho \frac{\partial {u}_{i}}{\partial {x}_{j}}\left(\frac{\partial {u}_{i}}{\partial {x}_{j}}+\frac{\partial {u}_{j}}{\partial {x}_{i}}\right)-\frac{2}{3}\rho \omega {\delta }_{ij}\frac{\partial {u}_{i}}{\partial {x}_{j}}$$

The set of constants $\phi$ is interpolated from the constants ${\phi }_{1}$ and ${\phi }_{2}$ for k–ω SST and the k–ϵ models respectively as stated by Menter^20^ using the following expression in which ${F}_{1}$ varies from 1 to 0 as it gets away from the wall and closer to the boundary layer edge:

5 $$\phi ={F}_{1}{\phi }_{1}+\left(1-{F}_{1} \right){\phi }_{2}$$

Additionally, the eddy-viscosity is defined as:

6 $${\nu }_{t}=\frac{{a}_{1}\kappa }{\mathrm{max}({a}_{1}\omega ;\Omega {F}_{2})}$$

where ${a}_{1}=0.3$ and $\Omega$ is the absolute value of the vorticity. Additionally, ${F}_{2}$ is defined as:

7 $${F}_{2}=\mathrm{tanh}\left({\mathit{max}\left(2\frac{\sqrt{\kappa }}{0.09\omega y};\frac{400\nu }{{y}^{2}\omega }\right)}^{2}\right)$$

The results produced by different k–ω SST turbulent model configurations will greatly vary in accuracy depending on the slant angle studied due to the complex effects of turbulence modelling. This is due to the fact that the flow around the Ahmed body and, subsequently, the flow around most ground vehicles is mainly governed by the turbulent wake bi-stability. The behavior of this wake depends greatly on the slant angle for the Ahmed body due to the strong pressure gradients over the slant. This is where the error introduced by the turbulence model comes into play, see Grandemande et al.^23^ and He et al.^24^. Different k–ω SST coefficients will produce better results for the different slant angle configurations as studied by Bounds et al.^25^. Additionally, the effect of different turbulence modelling methods on the prediction of the Ahmed body’s wake was studied by Guilmineau^26^, proving also that at different slant angles different turbulence modelling approaches produce more accurate predictions, among them the k–ω SST, which predicts drag with different accuracy for different slant angles. Nonetheless, the analysis of the effects of the different coefficients of the turbulence model on the CFD results is beyond the scope of this study, whose main focus is the development of the convolutional neural network. Therefore, the default k–ω SST coefficients have been used for the CFD simulations developed for this study. The reader must therefore bear this in mind when looking at the results provided in this study.

#### Validation

The CFD methodology was validated with experimental and computational data from Bayraktar et al.^13^ and Ahmed et al.^16^. For said validation the drag coefficient (Cd) was compared between the conducted simulations, experimental data, and the numerical results from the previously mentioned studies. To be able to ensure the independence of the drag force from the Reynolds number (Re), a high-enough Re of $4.4\times {10}^{6}$ was used for all validation simulations as proved by Bayraktar^13^.

The results (Cd) in Table 4 show good correlation with both experimental and previous CFD results for the Ahmed body geometries.

**Table 4:** Experimental and numerical simulation drag coefficient (Cd) comparison for an Ahmed body at 4.4 M Re for a range of slant angles and fixed leading radiuses of 100 mm.

Slant angle (°)	Results Cd (–)	Experimental Cd (–)
5	0.292	0.231
10	0.242	0.230
15	0.234	0.235
20	0.319	0.262
25	0.371	0.285
30	0.389	0.378
35	0.400	–

The slight error in drag force coefficient is understandable considering the following reasons for the discrepancies: first of all, these are two-dimensional simulations scaled to be able to obtain a three-dimensional drag coefficient, whilst both the experimental values obtained by Ahmed et al.^16^ are obtained through three-dimensional experiments and simulations. The fact, that this simulation is two-dimensional therefore, ignores one of the main drag producing phenomena, the counter-rotating vortex systems in the wake of the bluff body generated at the slant edges. These vortices main characteristic is their streamwise vorticity, which cannot be represented in 2-D models. Additionally, the difference in accuracy of the turbulence model for all the variations of slant angle has an important role in the produced error. As mentioned in “Turbulence model”, the accuracy of the results will depend highly on the turbulent model coefficients used which have been unaltered for the different slant angles. The turbulent wake behind these geometries will vary with the different configurations, which will introduce an error if the turbulence model coefficients are not adapted. On the other hand, the unsteady effects of the wake are also being neglected since the RANS turbulence models solve for an average steady-state solution which does not account for the oscillation in the results. Also, the cylindrical stilts used to hold the model in place during the conducted experimental test is not modelled in the 2-D geometry used for this study, and therefore, the drag contribution due to the stilts and their effect on the wake structure is not considered. It is also worth mentioning that the error increases rapidly as the slant angle increases. This is due to the increased adverse pressure gradient generated due to that angle. The turbulence model, although still more accurate than other models, underpredicts the separation point, to which the wake is really susceptible, and therefore making numerical simulations overpredict the drag force.

**Figure 4:**
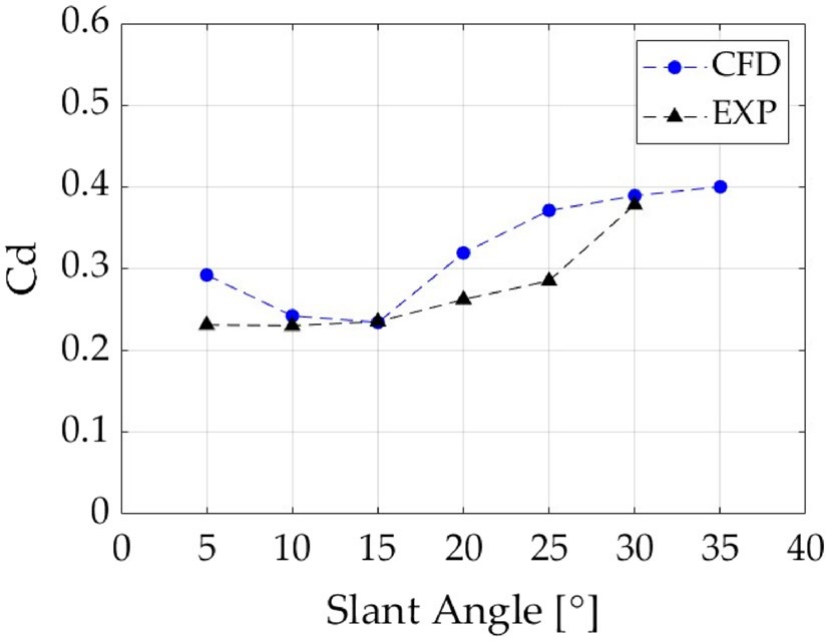
Drag coefficient (Cd) vs slant angle at $4.4\times {10}^{6}$ Re.

However, in spite of the slight error in drag prediction, the trends shown in both the experimental and the numerical studies are similar, as shown in Fig. 4. This proves that the simulations are indeed accurate enough to predict major flow-field characteristics and therefore can be used for training and testing of the CNN without having a major effect on the obtained results.

### Convolutional neural network

#### Input and output layers

Following the studies of Ribeiro et al.^7^ and Portal-Porras et al.^27^, in the present study three different input layers are used: a flow region channel (FRC) layer and two signed distance function (SDF) layers, one for the geometry of the vehicle and another one for the road.

**Figure 5:**
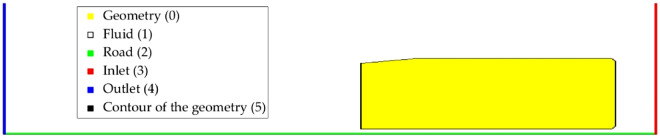
FRC representation of the domain.

The FRC layer provides information about the boundary conditions of the domain. In this layer a value is assigned to each cell of the grid depending on its boundary condition. In this case, the geometry is represented by a 0, the fluid by a 1, the road by a 2, the inlet by a 3, the outlet by a 4 and the contour of the geometry by a 5. Figure 5 shows an example of the FRC layer.

**Figure 6:**
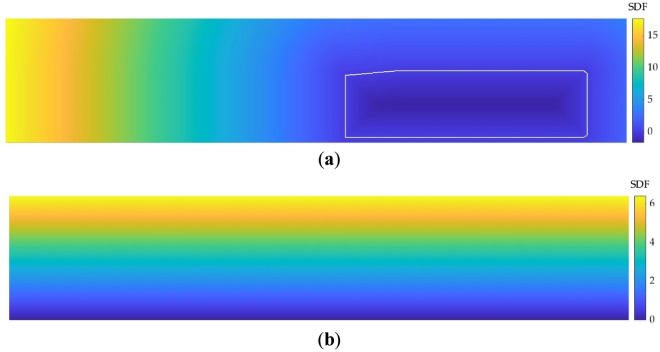
SDF representation. (**a**) SDF of the vehicle (the contour of the vehicle is marked in white); (**b**) SDF of the road.

SDF layers represent the distance between a cell and its nearest point on the contour of a defined geometry. As revealed in the study conducted by Guo et al.^28^, using SDF layers instead of the popular binary representation significantly improves the performance of the CNN. In this study two SDF layers are used, one for the vehicle and other one for the road. Two examples of these layers are provided in Fig. 6.

To create SDF layers, firstly the zero-level set ($Z$) is created, following Expression (1). The zero-level set consists of the cells where the contour of the analyzed geometry is located. In that cells the SDF is set to zero.

8 $$Z=\{\left(X,Y\right)\in {R}^{2}/ SDF\left(x,y\right)=0\}$$

where $(X, Y)$ are the coordinates of the cells of the geometry contour.

Then, the sign of SDF is defined depending on the location of the cell. If the cell is on the geometry contour, $SDF\left(x, y\right)=0$; if the cell is inside the geometry, $SDF\left(x,y\right)<0$; and if the cell is outside the geometry, $SDF\left(x,y\right)>0$.

Finally, after defining the sign, the SDF value of each cell is calculated by Expression (2).

9 $$SDF\left(x,y\right)=\underset{(X,Y)\in Z}{\mathrm{min}}\left|\left(x,y\right)-(X,Y)\right|\cdot sign$$

To generate the output layers, the velocity and pressure fields obtained with the CFD simulations are firstly interpolated to fit into a 256 × 512 grid. Then, the values of these fields are normalized following Expressions (3), (4) and (5).

10 $${u}_{x}^{*}=\frac{{u}_{x}}{{u}_{\infty }}$$

11 $${u}_{y}^{*}=\frac{{u}_{y}}{{u}_{\infty }}$$

12 $${p}^{*}=\frac{p}{\rho \cdot {u}_{\infty }^{2}}$$

where each variable marked with $*$ is a dimensionless variable.

After generating all the input and output layers, are ranged between 0 and 1 following Expression (6), in order to improve and speed up the training process.

13 $$ \Phi \prime  = \frac{{\Phi  - {\text{min}}(\Phi )}}{{{\text{max}}(\Phi ) - {\text{min}}(\Phi )}} $$

where Ф represents the values of each layer and Ф’ represents the ranged values.

#### CNN meshes

**Figure 7:**
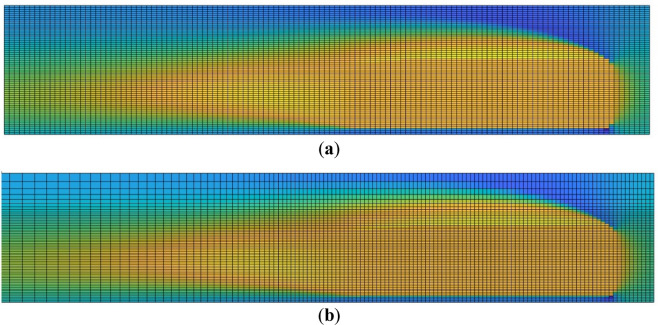
Meshes used in the CNN (not to scale). (**a**) Normal mesh; (**b**) refined mesh.

Compared to CFD methods, the main disadvantage of using neural networks is the limited resolution that they provide in the results. This is due to the flexibility provided by CFD tools to perform the meshing, allowing to generate cells of different sizes and shapes very simply; and to the computational limitations that CNNs have when performing the training, since the higher the resolution of the layers, the higher the computational cost. For that reason, in the present study an alternative CNN mesh is proposed, which is intended to resemble the mesh used in CFD simulations. In this alternative mesh, the resolution on the near-vehicle region is higher than in the outer region, and the cell size increases as the distance between the cells and the vehicle increases. In the normal mesh, the resolution of all the cells is of 5 cm × 2.5 cm; while, in the alternative mesh, the resolution of the cells located on the near-vehicle region is 4 cm × 2 cm. In both cases, the dimensions of the whole domain are 25.6 m × 6.4 m. Figure 7 provides an example of the proposed mesh.

#### CNN architecture

In this study, a CNN is designed and trained to predict the velocity and pressure fields around heavy vehicles. For designing and training the proposed network MATLAB 2022b^29^ commercial code with its Deep Learning Toolbox^30^ were used.

**Figure 8:**
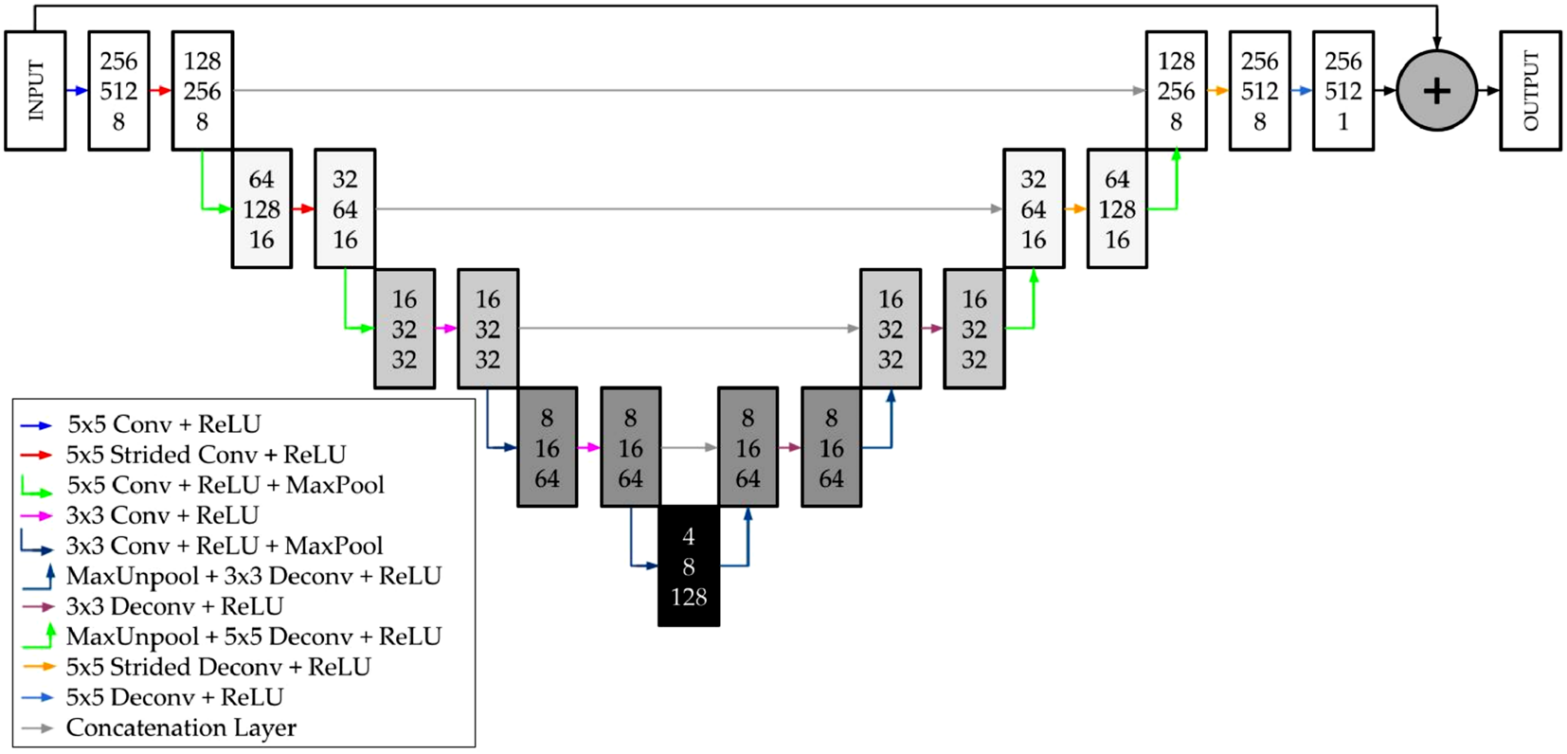
Schematic view of the proposed CNN.

The proposed CNN has a U-Net architecture^31^, which is a type of an encoder–decoder network. This network structure has been widely used for solving different fluid dynamics problems, due to its simplicity and generalization capacity, such as in Guo et al.^28^, Ribeiro et al.^7^, Kashefi et al.^32^ and Portal-Porras et al.^4^. The CNN used in this study contains four encoder/decoder blocks. Encoder blocks contain a convolutional layer, followed by a rectifier linear unit (ReLU) layer, and another convolutional layer follower by a ReLU layer and a Max Pooling layer. In the two first encoding blocks the kernel size is equal to 5, while in the last ones it is equal to 3. The number of filters is doubled after each encoding block. The decoding blocks, by deconvolution and Unpooling layers, perform the reverse process of their symmetrical blocks of the encoding phase. Encoding and decoding blocks are connected by concatenation layers. Figure 8 provides a diagram of the network. An individual decoder is trained for each magnitude, since, as demonstrated by Ribeiro et al.^7^, this architecture is the most accurate for solving fluid dynamic problems.

Adam optimizer^33^ is selected for training the network, with a batch size of 64, a weight decay of 0.005 and a learning rate of 0.001 that decreases as the training process progresses. Regarding the data-splitting ratio, data was randomly divided into 60% training, 30% validation and 10% testing.

## Results

In this section, the results obtained with CFD simulations and the predictions obtained with the CNN are compared, in order to assess the accuracy of the CNN. For this comparison, different aspects of the results are analyzed with the aim of identifying the main sources of error of the CNN. The cases analyzed throughout the section are the 22 belonging to the aforementioned test-set.

### Qualitative comparison

**Figure 9:**
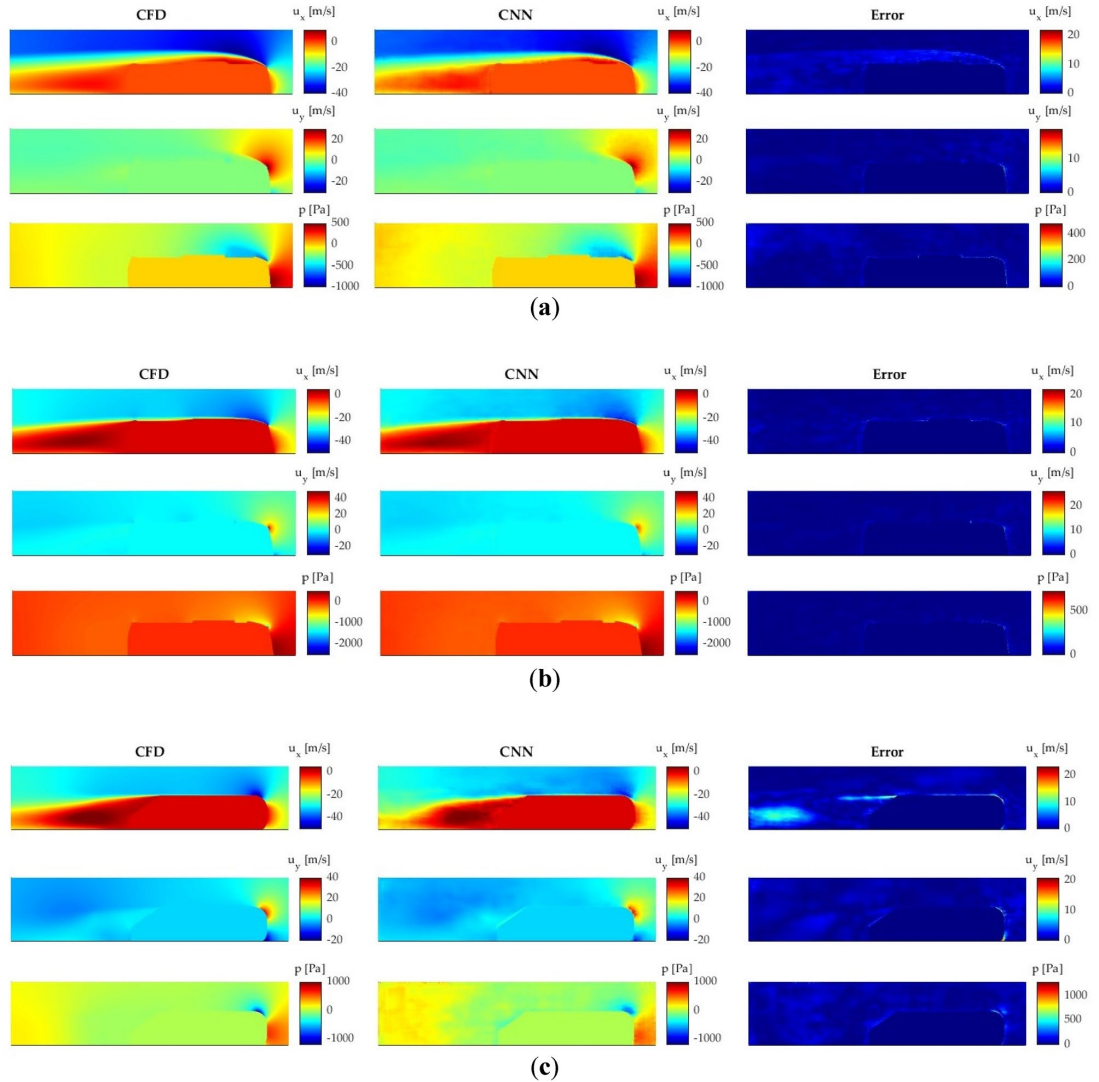
Comparison of the velocity and pressure fields obtained with CFD and CNN. (**a**) SC5; (**b**) SC7; (**c**) Ahmed body.

A qualitative comparison between the CFD and CNN predictions is performed in order to see the most troubleshooting locations. For this comparison, three different cases are considered, one for each main geometry. Figure 9a shows a SC5 case, Fig. 9b a SC7 case and Fig. 9c an Ahmed body case.

The results demonstrate that the CNN provides accurate predictions of the velocity and pressure fields around the proposed geometries. The region where the higher errors appear is the contour of the geometry, where the CNN is not able to correctly predict the boundary layer. Another troubleshooting area is the small wake generated at the topside of the SC5, whose size is underpredicted by the CNN, but the error is this region is low. Nevertheless, the CNN is able to reliably predict the flow characteristics, and all these mentioned errors are not considered significant. Additionally, the predictions of the wake behind the geometries, which is one of the most important regions for the aerodynamic study of vehicles, are very accurately.

The predictions of the Ahmed body show the largest errors in all the evaluated magnitudes, most markedly at the frontside of the geometry and at the wake behind. This may be attributed to two main reasons. The first one is the geometric difference between the Ahmed body and the rest of the geometries, and the second one that the cases of the Ahmed body are much less than those of the buses, so its incidence on CNN training is much lower.

### Velocity profiles

**Figure 10:**
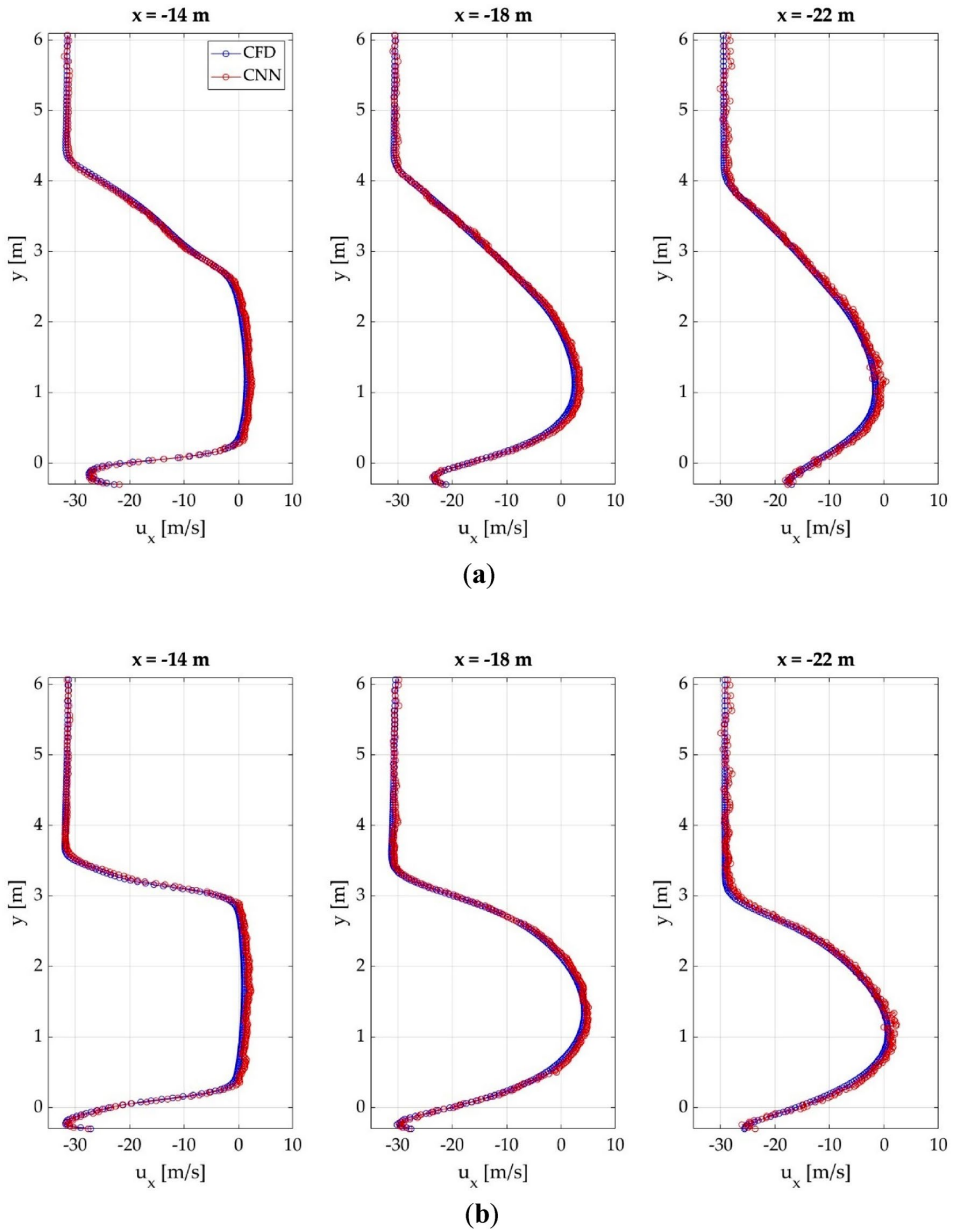

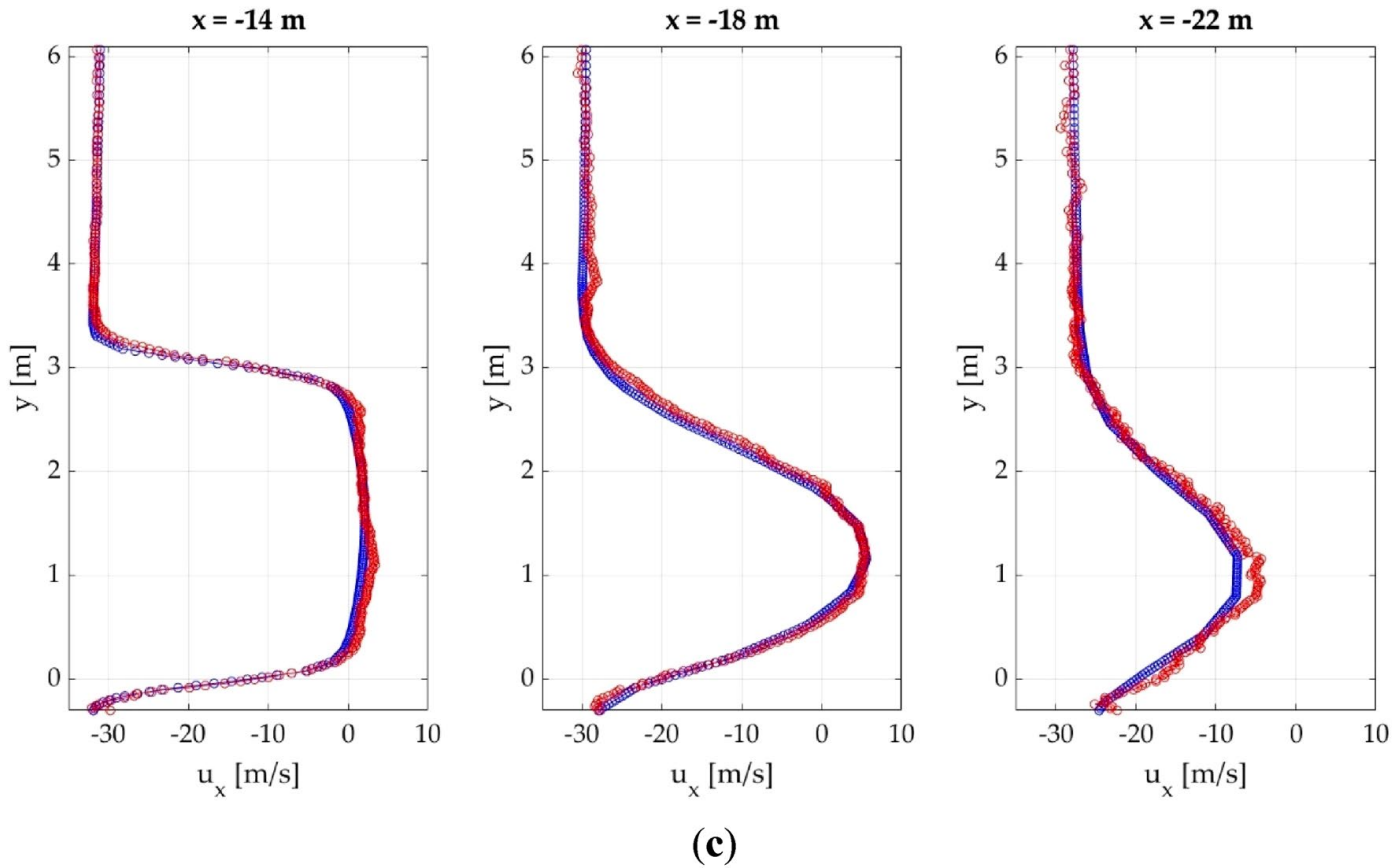
Comparison of the velocity profiles obtained with CFD and CNN at different locations from the front of the vehicle. (**a**) SC5; (**b**) SC7; (**c**) Ahmed body.

The velocity profiles on the wake behind the different cases in analyzed, in order to obtain detailed information about the accuracy of the CNN on that significant region. Figure 10 provides a comparison of the CFD and CNN results of the velocity profiles at different locations from the front of the vehicle for 3 different random cases.

The velocity profiles show two main regions, the lower region, influenced by the presence of the vehicle, and the upper region, which is very slightly influenced by the vehicle. The upper region remains constant for all the distances; but on the lower region, the horizontal velocity at is nearly 0 at the near wake, due to the influence of the vehicle, and increases as the distance from the vehicle increases. This is much more noticeable with the Ahmed body than with the SC5 and SC7.

The results demonstrate that the CNN is able to accurately reproduce this pattern for all the cases, following the same trend with very similar values. Table 5 summarizes the maximum absolute error for each vehicle at different distances.

**Table 5:** Maximum absolute error of the velocity profiles for each case at different distances.

Distance from the front (m)	SC5 (m/s)	SC7 (m/s)	Ahmed body (m/s)
14	1.798	2.081	3.321
16	1.086	1.301	2.478
18	1.275	1.945	2.381
20	2.076	1.425	2.84
22	2.018	1.962	3.131

The larger differences appear with the Ahmed body, but as explained previously, these differences can be attributed to the geometrical difference between the Ahmed body and the other cases. Nevertheless, the maximum absolute errors are considered acceptable.

### Data distribution

**Figure 11:**
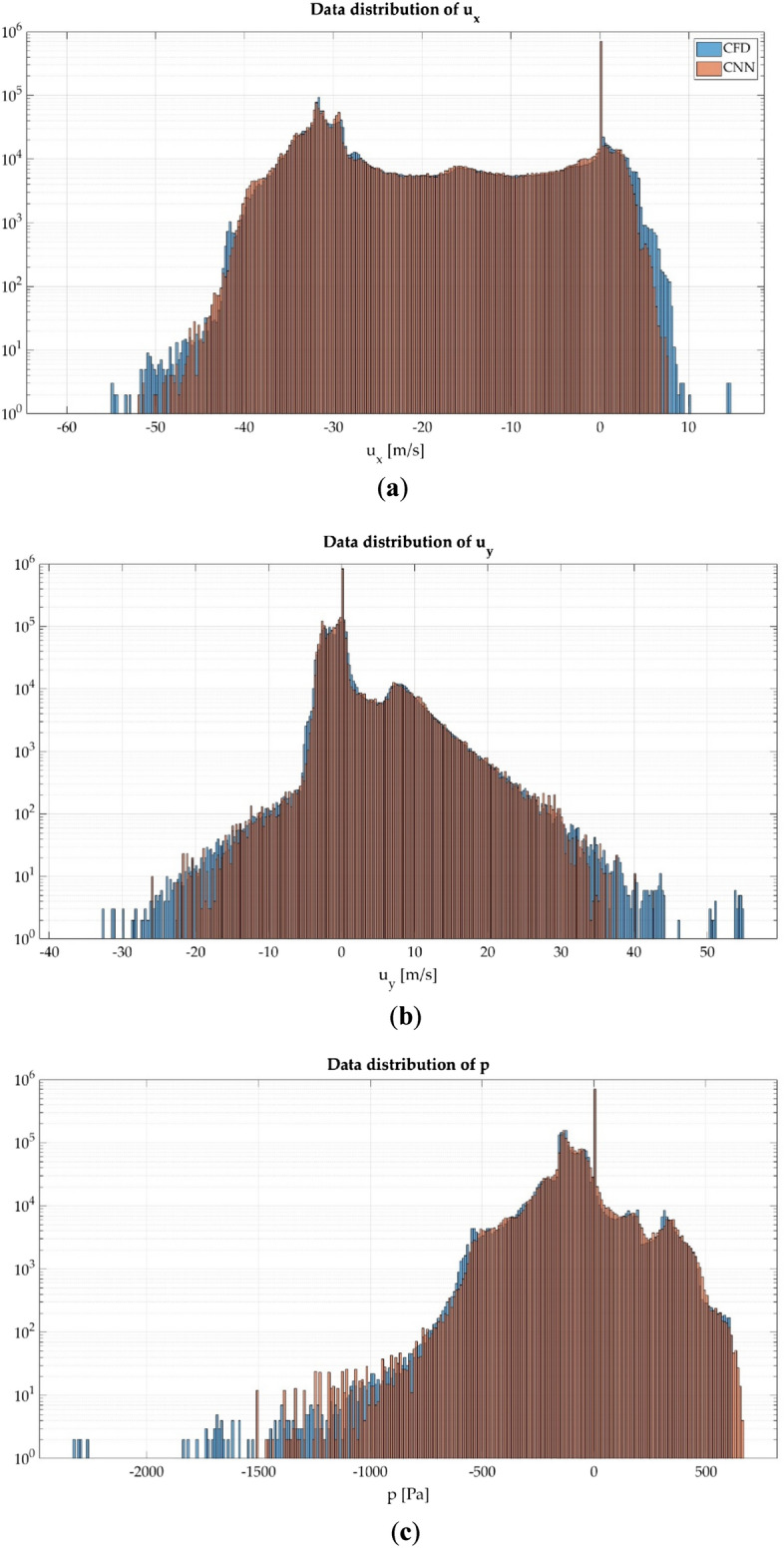
Data distribution of the analyzed magnitudes. (**a**) ${u}_{x}$; (**b**) ${u}_{y}$; (**c**) $p$.

The data distribution of each of the analyzed magnitudes is studied in order to perform a quantitative comparison of the obtained results. Figure 11a shows the data distribution of ${u}_{x}$, Fig. 11b of ${u}_{y}$ and Fig. 11c of $p$.

The data distribution histograms show that both CFD and CNN follow a nearly equal trend. The largest differences between both methods appear in the values of the extremes, since the CNN underpredicts these values, most notably with the velocity fields.

Additionally, the arithmetic mean (µ) and standard deviation (σ) of the results obtained with both methods is calculated from these histograms. Table 6 shows the values of µ and σ. In accordance with the histograms the arithmetic mean and standard deviation is similar in all the analyzed magnitudes.

**Table 6:** Arithmetic mean and standard deviation of the analyzed magnitudes.

Method	CFD			CNN		
	${u}_{x}$	${u}_{y}$	$p$	${u}_{x}$	${u}_{y}$	$p$
Arithmetic mean (µ)	− 16.4194	0.5836	− 78.3732	− 16.3831	0.6345	− 75.0606
Standard deviation (σ)	14.725	3.9418	142.5327	14.697	4.0044	140.3397

### Error analysis

Aiming to compare the accuracy of the used CNN meshes, the errors obtained with each one for predicting the whole test-set are evaluated. For this comparison, the maximum and mean errors are considered. For the maximum error (${\varepsilon }_{max}$), the average value of the maximum error achieved with all the test-set samples is calculated. As in the alternative mesh the cells have different sizes, to calculate the mean error (${\varepsilon }_{mean}$) Expression 7 is considered, in which the error of each cell is weighted by its area. Table 7 shows the results of maximum and mean error obtained with each mesh.

14 $${\varepsilon }_{mean}=\frac{1}{N}\sum_{n=1}^{N}\frac{\sum_{cell=1}^{512\times 256}{\varepsilon }_{cell}\cdot {A}_{cell}}{{A}_{mesh}}$$

where N represents the cases of the test-set, ${A}_{cell}$ the area of each cell and ${A}_{mesh}$ the total area of the mesh.

**Table 7:** Maximum and mean error of the analyzed magnitudes.

Error	Normal mesh			Alternative mesh		
	${u}_{x}$	${u}_{y}$	$p$	${u}_{x}$	${u}_{y}$	$p$
Maximum error (${\varepsilon }_{max}$)	23.1401	22.2546	665.5008	22.2956	16.7331	642.4196
Mean error (${\varepsilon }_{mean}$)	0.4768	0.2762	9.9947	0.4324	0.251	10.3046

The results demonstrate that the alternative mesh is able to reduce both the maximum and mean errors in all the cases, except for the mean error of the pressure field. This is because, as shown in the qualitative comparison, the boundary layer is the main source of errors. As the alternative mesh has a higher mesh resolution in that area, and therefore, a higher accuracy, it is able to reduce error that region.

### Performance comparison

As the main objective of using data-driven methods instead of CFD methods for predicting flow physics is to reduce the computational time, in this section a comparison of the computational time required by each method to perform the predictions is conducted. Table 8 shows this comparison. In both cases a single Intel Xeon Gold 5120 CPU core was used.

**Table 8:** Computational time required by each method to obtain the predictions of velocity and pressure fields.

Method	Time [s]	Speedup
CFD	27,528.21	–
CNN	2.57	10,711.37

CFD simulations required an average of 27,528.21 s to obtain high-fidelity results with low residuals. In contrary, the CNN required 56.46 s to predict the velocity and pressure fields of the 22 geometries of the test set, which means that an average of 2.57 s is required to obtain the predictions of a single geometry. Therefore, the CNN proposed in this study is 10,711 times faster than CFD simulations. Nonetheless, the training of the proposed networks required about 24 h, using the previously-mentioned hardware.

## Conclusions

In the present article, CFD simulations to analyze the aerodynamics of different bus geometries were conducted. With the obtained results, a CNN for velocity- and pressure-field prediction around the mentioned geometries was trained, with the objective of assessing the capacity of the CNN to reproduce the flow fields obtained with CFD tools. Since this study is focused on the suitability of the CNN to study the potential of CNN based ML methods for prediction ground vehicle aerodynamic characteristics, basic CFD methods and models have been employed, in order to reduce computational resources and time. Additionally, as one of the main disadvantages of DL-based methods with respect to CFD is the limited flexibility they provide to represent the computational domain with an appropriate meshing, an alternative meshing technique for the CNN is proposed, which tries to resemble the meshes used in CFD simulations.

The velocity and pressure fields demonstrate that the CNN is able to accurately predict the flow phenomena around the buses. The flow fields show that the most troubleshooting area for the CNN is the boundary layer, showing the largest errors in that region. Nevertheless, in the wake behind the vehicles, which is the most affected area by the diffuser modifications, the predictions are accurate, showing low errors, except for the Ahmed body where these errors are slightly larger. Even so, since the geometry of the Ahmed body is markedly different from the other geometries used in training, and errors are relatively low, this is not considered a problematic case.

The data distribution histograms show that the results obtained with both methods are similar, in both values and trend. This analysis shows that, for all the evaluated magnitudes, the CNN does not properly predict the values located at the extremes of the data distribution. However, in the most repeated values, the data distribution is nearly equal with both methods.

Regarding the CNN mesh comparison, the mesh proposed for CNN performance enhancement is able to reduce both the maximum and mean errors. As shown in the qualitative comparison, the maximum errors appear in the boundary layer. As the resolution of the proposed mesh is higher in this region, the predictions are more accurate, reducing the error. Although in the areas farther away from the geometry the resolution is worse, as in these areas the influence of the geometry is less significant, and therefore, more generalizable for all cases, in that area the errors remain almost equal. This implies a reduction of the mean and maximum error.

As for computational time, the CNN reduces the computational time required to obtain the results in four orders of magnitude, using the same hardware. These results are within the expected range, given that the main reason for using data-driven methods instead of the traditional numeric methods is the substantial reduction of computational time and resources that this kind of methods entail.

Nevertheless, the current neural network model presents some limitations regarding the domain and boundary layer modelling. On one hand, the domain resolution is limited to two dimensional simulations or coarse meshes and, in the other hand, the predictions within the boundary layer are improvable.

## Data Availability

The datasets used and/or analysed during the current study available from the corresponding author on reasonable request.
